# Significantly Improving the High-Temperature Tensile Properties of Al_17_Cr_10_Fe_36_Ni_36_Mo_1_ Alloys by Microalloying Hf

**DOI:** 10.3390/ma16216836

**Published:** 2023-10-24

**Authors:** Zhihua Chen, Jianbin Wang, Yuhao Jia, Qingfeng Wu, Xiaoming Liu, Linxiang Liu, Junjie Li, Feng He, Zhijun Wang, Jincheng Wang

**Affiliations:** State Key Laboratory of Solidification Processing, Northwestern Polytechnical University, Xi’an 710072, China; 239987149@mail.nwpu.edu.cn (Z.C.);

**Keywords:** dual-phase high-entropy alloys, hafnium, microstructure, high-temperature properties, microalloying

## Abstract

Dual-phase high-entropy alloys with excellent room temperature and high-temperature properties have been widely studied as potential high-temperature structural materials. However, interface weakening causes its high-temperature performance to decline at higher temperatures, severely limiting further development. In this study, a series of Al_17_Cr_10_Fe_36_Ni_36_Mo_1_Hf_x_ (x = 0, 0.03, 0.15, 0.3, 0.5, and 0.8 at%) alloys were prepared to study the effect of Hf content on the microstructure and mechanical properties of the matrix alloy. The results indicate that with the addition of the Hf, the Hf-rich phase began to precipitate at the interface and inside the B2 phase in the matrix alloy. In contrast, the morphology of both the FCC and B2 phases had no noticeable change. With the increase in Hf content, the high-temperature strength and ductility of the alloy first increased and then decreased, while the room temperature performance remained almost unchanged. Benefiting from the hindrance of the Hf-rich phase to grain boundary sliding and dislocation movement during high-temperature deformation, the tensile strength, yield strength, and plasticity of the matrix alloy increased from 474 MPa, 535 MPa, and 8.7% to 816 MPa, 923 MPa, and 42.0% for the Al_17_Cr_10_Fe_36_Ni_36_Mo_1_Hf_0.5_ alloys, respectively. This work provides a new path for designing a high-entropy alloy with excellent high-temperature mechanical properties.

## 1. Introduction

Developing alloys with ultrahigh strength and excellent ductility simultaneously over a wide temperature range is crucial for developing high-temperature steam turbines [1,2,3]. Recently, as a category of high-entropy alloys (HEAs), dual-phase high-entropy alloys (DP-HEAs) with a soft FCC phase and hard BCC (B2) phase have been reported to have excellent strength–ductility synergy at ambient temperature and high temperature [4,5,6,7,8,9,10,11,12,13], and thus have great potential to be used as steam turbine hot end components. Unfortunately, due to the rapid softening of the phase with the BCC structure at high temperatures, the yield strength of the alloy will significantly decrease [10,14,15,16,17,18]. Moreover, due to the weakness of grain boundaries and phase boundaries, the cracks initiate rapidly and propagate along the interface during high-temperature deformation, severely limiting the high-temperature application of FCC/B2 dual-phase high-entropy alloys [19,20,21,22,23].

As we all know, the alloying of metals with certain specific elements can very effectively improve alloy properties through various strengthening methods such as precipitation strengthening, solution strengthening, and interface strengthening [24,25,26,27]. Hf is an essential alloying element for both the interface strengthening of superalloys and the strengthening of the Ni-Al B2 phase [28,29,30,31,32,33]. Recently, some studies have shown that adding a small amount of Hf to a single-phase FCC high-entropy alloy could improve the room-temperature mechanical property of the alloy without significantly reducing the ductility of the alloy through the precipitation of intermetallic compounds such as Ni_7_Hf_2_ and Laves phases [34,35]. However, these studies mainly focus on single-phase high-entropy alloys, and the addition of Hf is relatively large, usually more than one atom. Therefore, the effect of trace Hf addition on the properties of the alloy at room temperature and high temperature is worthy of in-depth investigation.

In this paper, we designed and prepared Al_l7_Cr_10_Fe_36−x_Ni_36_Mo_1_Hf_x_ dual-phase high-entropy alloys (x = 0, 0.03, 0.15, 0.3, 0.5, and 0.8 in at%) with FCC and B2 phases. Firstly, the same thermal and mechanical treatment was conducted on the Al_l7_Cr_10_Fe_36−x_Ni_36_Mo_1_Hf_x_ cast alloy to eliminate segregation and solidification defects in the cast alloy. Then, a systematic study was conducted on the effect of Hf content on the microstructure and mechanical properties of the Al_l7_Cr_10_Fe_36−x_Ni_36_Mo_1_Hf_x_ alloy. The addition of Hf can simultaneously improve the high-temperature strength and ductility of the matrix alloy in the temperature range from 600 °C to 900 °C, especially at 700 °C. The strengthening and toughening mechanisms at 700 °C in the Al_l7_Cr_10_Fe_35.5_Ni_36_Mo_1_Hf_0.5_ alloys were analyzed by comparing the differences in fracture behavior and microstructure between the Al_17_Cr_10_Fe_36_Ni_36_Mo_1_ and Al_17_Cr_10_Fe_35.5_Ni_36_Mo_1_Hf_0.5_ alloys.

## 2. Experiment

### 2.1. Materials Preparation

Alloys with a nominal composition of All7Cr10Fe36-xNi36Mo1Hfx (at%) were prepared by arc-melting high-purity raw metals (>99.9 wt%) in a Ti-gettered high-purity Ar atmosphere. When the values of x are 0, 0.03, 0.15, 0.3, 0.5, and 0.8, alloys are denoted as Hf0, Hf0.03, Hf0.15, Hf0.3, Hf0.5, and Hf0.8, respectively. The ingots were flipped over and remelted at least five times for chemical homogeneity and then rapidly dropped into a water-cooled copper mold with dimensions of 100 × 12 × 5 mm^3^. The five as-cast ingots were homogenized at 1200 °C for 8 h to reduce solute segregation. Then, the samples were thermomechanically treated using the phase-selective recrystallization (PSR) method [7]. For the PSR process, the homogenized alloy was cold-rolled by 15% and crystallized at 1200 °C for 20 min for two cycles, followed by 30% cold-rolling and crystallized at 1200 °C for 20 min. Finally, the five PSR alloys were aged at 700 °C for 12 h. Air cooling was adopted in all the heat treatment procedures to avoid cracks.

### 2.2. Mechanical Tests and Materials Characterizations

Uniaxial tensile tests were performed at 25 °C, 600 °C, 700 °C, 800 °C, and 900 °C in air with a constant strain rate of 1 × 10^−3^ s^−1^. Flat dog-bone-shaped specimens with a gauge length of 12 mm and a cross-sectional dimension of 3 × 2 mm^2^ were used. An extensometer was employed to monitor the strains for room-temperature tensile tests directly. The microstructure of the five annealed alloys was characterized by a field-emission scanning electron microscope (SEM, TESCAN MIRA3, TESCAN, Brno-Kohoutovice, Czech) equipped with energy dispersive spectroscopy (EDS, OXFORD, Abingdon upon Thames, UK) detectors and an optical microscope (OM, OLYMPUS OLS4000, OLYMPUS, Tokyo, Japan). Transmission electron microscopy (TEM, Talos F200X, FEI Czech, Brno-Královo Pole, Czech) was used to characterize the precipitates and elemental distribution in the Hf0.5 alloys. Electron backscattered diffraction (EBSD) characterizations were performed using an Oxford Instrument detector (OXFORD, UK). The rectangular specimens were prepared by electrospark wire-electrode cutting and then polished with 240-, 800-, 1500-, and 2500-grit SiC paper to remove the oxide layer. The polished specimens used in microstructure characterization were electron-polished in a mixed solution of 10% perchloric acid and 90% absolute ethanol with a direct voltage of 30 V for 6 s at room temperature. For the EBSD characterizations, the polished specimens were subsequently polished with silica suspension and then ultrasonically cleaned in ethanol for 5 min. The TEM specimens were mechanically ground down to ~50 μm thickness before being electrolytically double sprayed. The electrolytic double spray process is carried out through MTP-1A electrolytic double spray thinning apparatus at −20 °C in corrosive fluids containing 10% perchloric acid by volume and 90% alcohol by volume. The range of current and voltage is 50–70 mA and 40–50 V, respectively.

## 3. Results and Discussion

### 3.1. Microstructure and Element Distribution Characterizations

**Figure 1:**
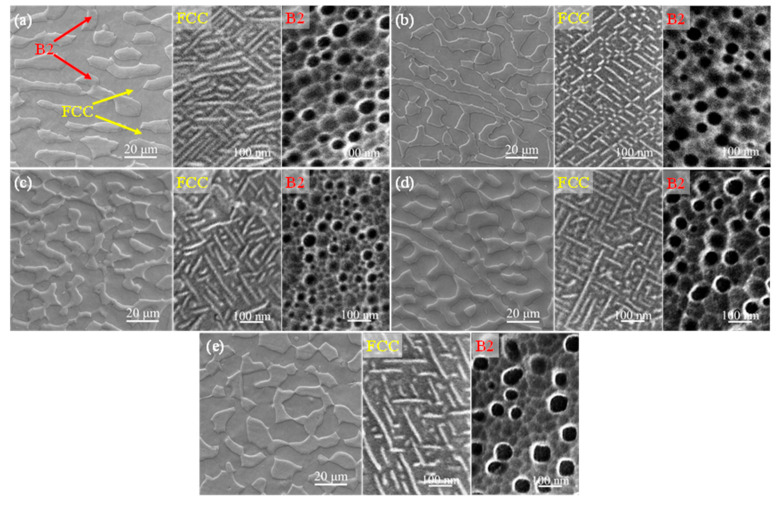
SEM images at different magnifications of (**a**) Hf0 alloy, (**b**) Hf0.03 alloy, (**c**) Hf0.15 alloy, (**d**) Hf0.3 alloy, and (**e**) Hf0.5 alloy. These five alloys were composed of the FCC and B2 phases. SEM images at high magnification of the FCC phase and B2 phase in the corresponding alloys are placed on the right side of each image.

The microstructures of the five alloys prepared in the current work are shown in Figure 1. With an increasing Hf content, there was no significant change in the morphologies of both the FCC and B2 phases. Meanwhile, from the high-magnification SEM images displayed in Figure 1, both nanoscale spherical precipitates with a size of 10~90 nm and needle-shaped precipitates with a width of 10~20 nm arranged regularly were observed in these five alloys distributed in the inner B2 phase and FCC phase, respectively. The nanostructure inside these two phases was also very similar. Therefore, adding a small amount of Hf had no significant effect on the microstructure of the alloys.

**Figure 2:**
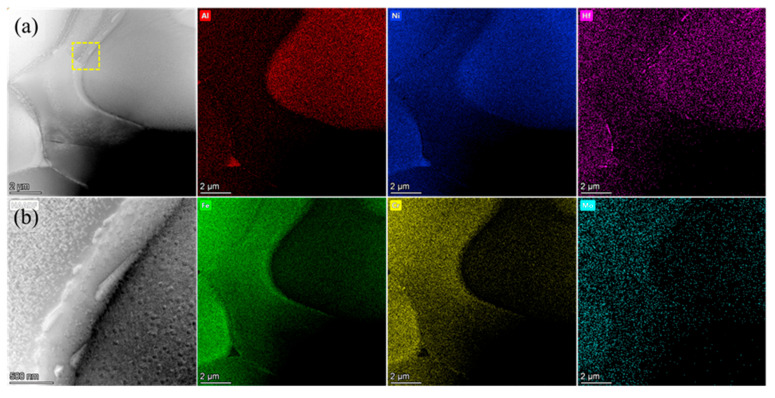
Transmission electron microscopy and energy dispersive spectroscopy analysis for the Hf0.5 alloys. (**a**) High-angle annular dark-field (HAADF) images of the Hf0.5 alloys and the corresponding area EDS images. (**b**) High-magnification HAADF images of the region marked in (**a**).

To reveal the distribution of Hf in alloys, TEM analysis was conducted on the Hf0.5 alloy with the highest Hf content, and the results are shown in Figure 2. Figure 2a shows the high-angle annular dark-field (HAADF) image of the Hf0.5 alloys and the corresponding EDS area scanning map. Based on previous investigations [36,37], the region enriched in Ni and Al was the B2 phase, while the phase enriched in Fe and Cr was the FCC phase. Mo was mainly distributed in the FCC phase and was relatively poor in the B2 phase. It can be observed that there were some Hf-rich precipitates at the phase boundary and grain boundary of the FCC phase. From Figure 2b, the Hf-rich phase at the phase boundaries appears as needle-shaped structures, with lengths ranging from 120 to 760 nm. In addition to the enrichment of Hf in the precipitates at the interface, Hf was uniformly distributed in the FCC and B2 phases.

**Figure 3:**
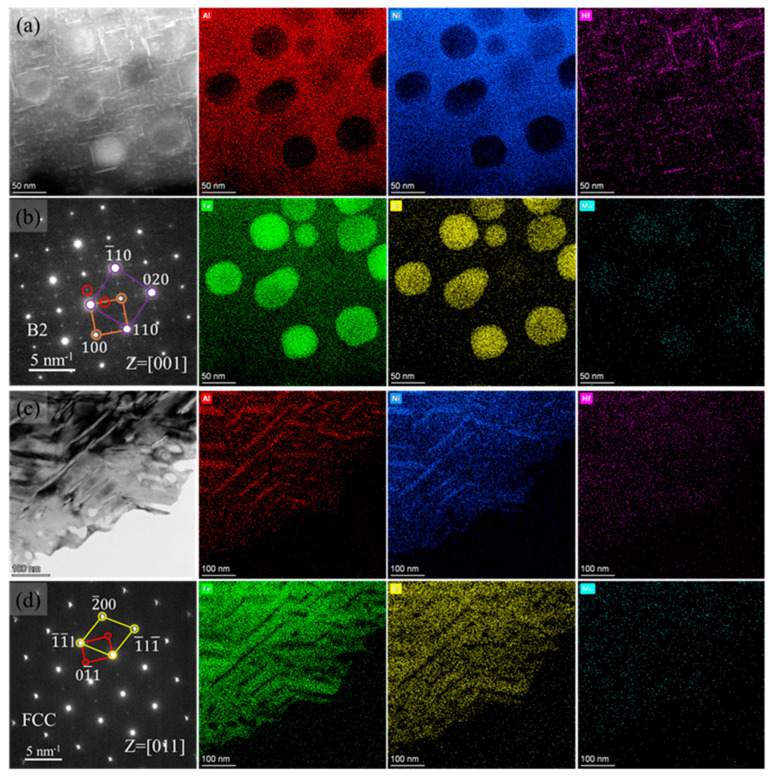
TEM images of the precipitations inside the B2 phase and FCC phase in Hf0.5 alloy. (**a**) HAADF images of the B2 phase and EDS images of the corresponding area, (**b**) selected area electron diffraction (SAED) patterns in the B2 phase. (**c**) HAADF images of the FCC phase and EDS images of the corresponding area, (**d**) SAE D patterns in the FCC phase.

Figure 3 shows the precipitates inside the B2 and FCC phases in the Hf0.5 alloy. The HAADF images of the B2 phase in Figure 3a indicate that there were two types of precipitates: large spherical precipitates and fine acicular precipitates. According to the EDS profile in Figure 3a, it can be inferred that the spherical precipitates enriched in Fe, Cr, and Mo are BCC phases, while the matrix phase enriched in Ni and Al is the B2 phase [36]. The diameter of the spherical particle was measured to be 33–72 nm, consistent with the above SEM results. It is worth noting that Mo, mainly distributed in the BCC phase, was almost insoluble in the B2 phase, in accordance with a previous report [38]. The acicular precipitates with widths of 1~5 nm interlaced with each other and were rich in Hf. In addition, Hf is preferably distributed in the B2 phase rather than the BCC phase (Figure 3a). The SAED patterns shown in Figure 3b also confirm that there were two types of precipitates in the B2 matrix and illustrate the superlattice diffraction of the B2 phase, as marked by the orange circle. Moreover, diffraction spots of the BCC and Hf-rich phases are also observed in Figure 3b, as marked by the purple and red circles, respectively.

A similar characterization was conducted on the FCC phase, and the results are shown in Figure 3c,d. From the HAADF images of the FCC phase, it can be seen that there were many needle-shaped structures with a width of 6~20 nm with a staggered arrangement in the matrix, in accordance with previous SEM results (Figure 1e). In addition, according to the corresponding EDS images and SAED pattern (Figure 3c,d), it can be inferred that these needle-like structures precipitated in the matrix were the L1_2_ phases and that the matrix was the FCC phase. These needle-shaped structures were mainly composed of Ni and Al, while the matrix phase mostly consisted of Fe and Cr. Both Mo and Hf were evenly distributed in these two phases. Therefore, after the addition of a small amount of Hf (0.5 at%) in the alloy, the precipitation of the Hf-rich phase occurred at the grain boundaries and the phase boundaries, as well as the interior of the B2 phase.

### 3.2. Mechanical Properties at Room Temperature and High Temperature

**Figure 4:**
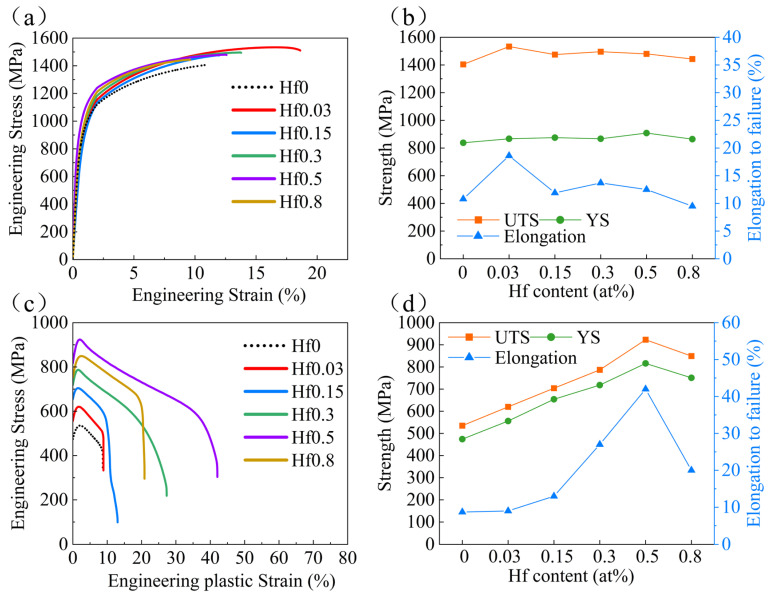
Mechanical properties of the designed alloys at 25 °C and 700 °C. Tensile engineering stress-strain curves of the samples tested at (**a**) 25 °C and (**c**) 700 °C. Variation in ultimate tensile strength, yield strength, and elongation to failure of the samples as a function of Hf content at (**b**) 25 °C and (**d**) 700 °C.

**Figure 5:**
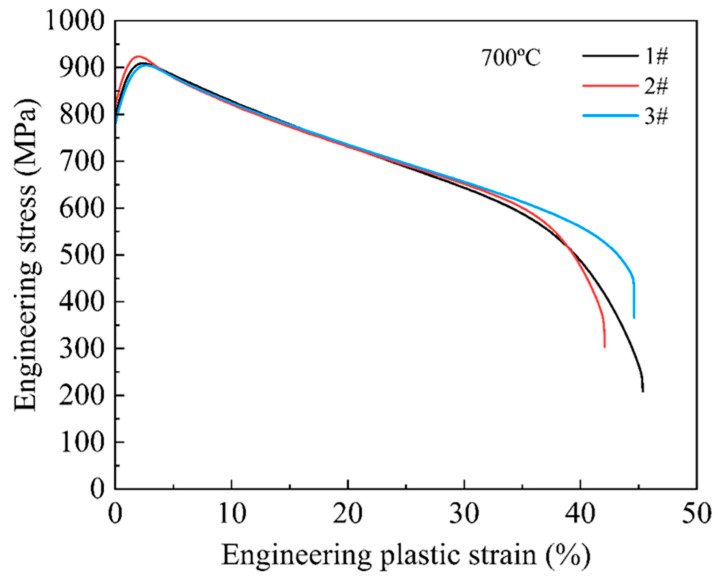
Tensile engineering stress-strain curves of the Hf0.5 alloy tested three times at 700 °C.

The mechanical properties tested at room temperature and 700 °C of the six alloys with different Hf contents are shown in Figure 4, and the corresponding specific results of the mechanical properties are summarized in Table 1. From Figure 4a, it can be seen that all six alloys exhibited a high yield strength (above 830 MPa) and ultimate tensile strength (above 1400 MPa), as well as good tensile plasticity (above 9%), which was related to the hierarchical microstructure formed in the alloys (Figure 1). It is worth noting that with the increase in Hf content, the mechanical properties of the alloy at room temperature remained basically unchanged. Interestingly, the addition of Hf did not harm the plasticity of the alloy (Figure 1), which may be related to the low Hf content. The tensile engineering stress-strain curves tested at 700 °C for the six alloys are displayed in Figure 4c. Excitingly, the addition of Hf in the matrix alloys will significantly improve the mechanical properties. In Figure 4d, with the increase in Hf content, both the strength and ductility of the alloy significantly increased and then decreased. The Hf0.5 alloys with the best high-temperature mechanical properties demonstrated a yield strength of 817 MPa, ultimate tensile strength of 923 MPa, and ductility of 42%, realizing an excellent combination of strength and ductility. In order to confirm the repeatability of the high-temperature tensile test of the alloy, the high-temperature tensile test of Hf0.5 alloy, which has the best high-temperature performance, was repeated three times at 700 °C. The results of the three repeated tests are shown in Figure 5, and the specific results of the corresponding performance are summarized in Table 2. As shown in Table 2, the high-temperature tensile test repeatability of the alloy is good.

**Figure 6:**
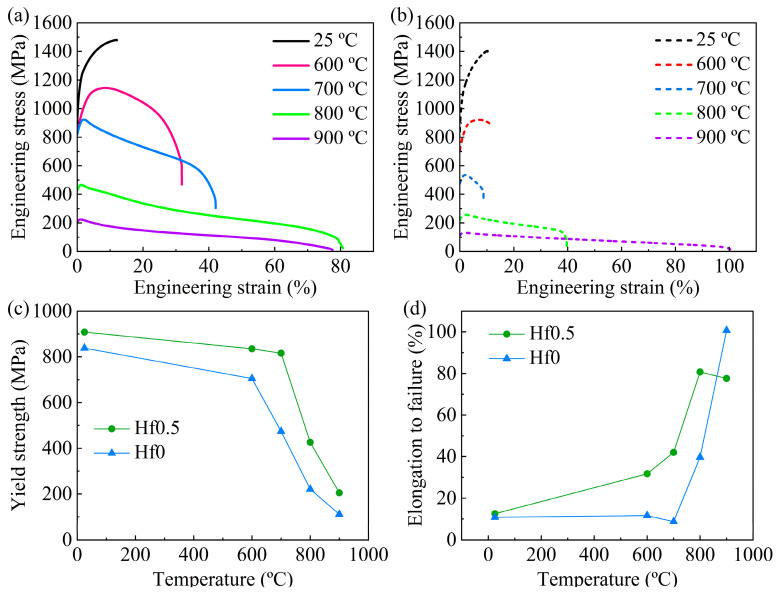
Tensile engineering stress-strain curves of the (**a**) Hf0.5 alloy and (**b**) Hf0 alloy tested from 25 °C to 900 °C. The variations in the yield strength and fracture strain of the Hf0.5 alloy and Hf0 alloy as a function of temperature are shown in (**c**) and (**d**), respectively.

To further reveal the strengthening effect of Hf on the alloy at different temperatures, the high-temperature mechanical properties of Hf0.5 alloys were tested at 600 °C, 800 °C, and 900 °C, and the tensile engineering stress-strain curves are shown in Figure 6a. In addition, the Hf0 alloys were also tested at the same temperature for comparison. The extracted tensile yield strength (σ_YS_), ultimate tensile strength (σ_UTS_), and fracture elongation (ε_f_) of these two alloys tested in the temperature range from 25 °C to 900 °C are tabulated in Table 3. As shown in Figure 6c, as the temperature increased, the yield strength of both the Hf0 alloys and Hf0.5 alloys exhibited a trend of slowly descending first and then rapidly descending. Although the variation in the yield strength was consistent in these two alloys, there were some differences in the specific details. For the Hf0 alloys, the yield strength decreased gradually from 838 MPa to 705 MPa as the temperature increased from 25 °C to 600 °C, but when the temperature increased to 700 °C, the yield stress of the alloys dropped dramatically to 474 MPa. This indicates that the Hf0 alloy had undergone significant softening at 700 °C, related to the softening of the B2 phase in the alloys at high temperatures [39]. When the deformation temperature reached 800 °C, the Hf0.5 alloy began to soften. In addition, the Hf0.5 alloys exhibited a higher strength than the Hf0 alloys at all the tested temperatures, especially at high temperatures. This indicates that Hf is highly effective in improving the high-temperature performance of high-entropy alloys. Figure 6d displays the variation in the fracture strain of the Hf0.5 and Hf0 alloys as a function of temperature. With an increasing temperature, the fracture strain of the Hf0 alloys exhibited a trend of first decreasing and then increasing. The Hf0 alloys had a minimum fracture strain at 700 °C. In contrast, the fracture strain of the Hf0.5 alloys significantly increased as the temperature increased.

Based on the above analysis, it can be found that the Hf0.5 alloy exhibited a higher softening temperature than the Hf0 alloys, which was related to the Hf-rich phase in the Hf0.5 alloy (Figure 2 and Figure 3). First, as shown in Figure 2a, the Hf-rich phases at grain and phase boundaries in the Hf0.5 alloys would pin grain and phase boundaries to hinder their sliding during high-temperature deformation. Then, the needle-shaped Hf-rich phase with a length of less than 50 nm distributed in the B2 phase (Figure 3a) hindered the movement of dislocations at high temperatures.

### 3.3. Fracture Behavior of Hf0.5 Alloy at 700 °C

**Figure 7:**
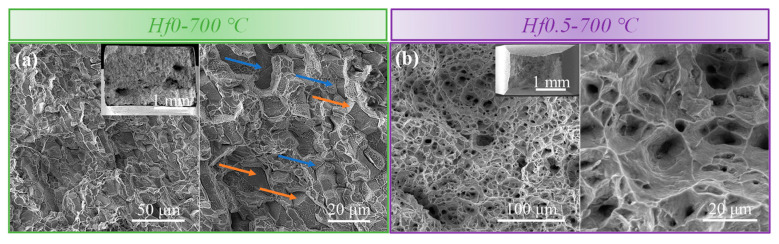
Secondary electron micrographs of the fracture morphologies of the alloys after tensile tests at 700 °C: (**a**) the Hf0 alloy and (**b**) the Hf0.5 alloy. The blue and orange arrows represent intergranular cracks and intergranular planes, respectively.

The elongation to failure at 700 °C of the Hf0.5 alloys was five times higher than that of the Hf0 alloy as shown in Figure 6d, meaning there were different fracture behaviors in these two alloys. To analyze the difference in failure and crack mechanisms between the Hf0.5 alloys and the Hf0 alloys at 700 °C, the fracture morphologies of the two alloys after tensile tests at 700 °C were characterized by multiple characterization techniques and are displayed in Figure 7. For the Hf0 alloys, obvious grain facets (orange arrow) and interface cracks (blue arrow) were observed on the fracture surface, as shown in Figure 7a, revealing the brittle intergranular fracture mode. In contrast, the fracture surface of the Hf0.5 alloys was filled by dimples, which revealed the ductile transgranular fracture mode, as shown in Figure 7b. These results indicate that the addition of Hf in the alloy transformed the fracture mode from an intergranular fracture to transgranular fracture.

**Figure 8:**
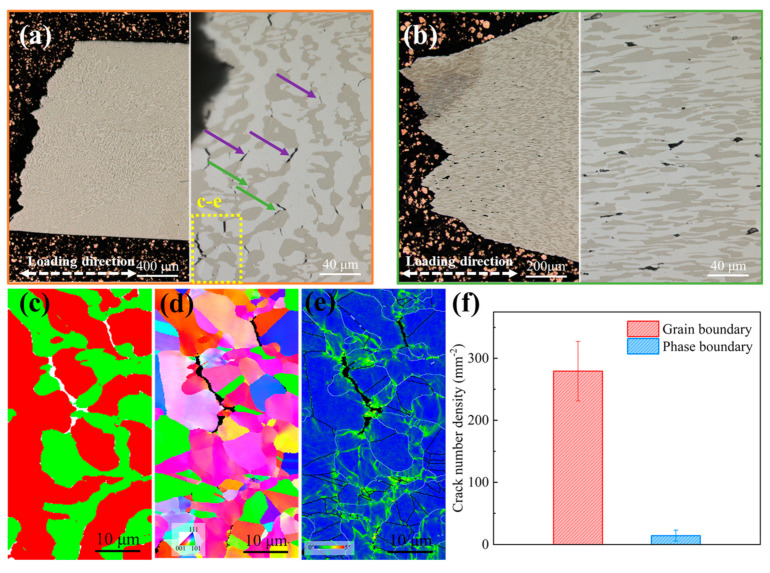
Failure characteristics of the Hf0 and Hf0.5 alloys. Optical micrographs of the lateral surface of the fractured alloys tensioned at 700 °C: (**a**) Hf0 alloy (**b**) and Hf0.5 alloy. (**c**) Phase map, (**d**) inverse pole figure (IPF) map, and (**e**) kernel average misorientation (KAM) map of the region masked by the yellow dotted line in the fractured Hf0 alloy. The red and green areas in (**c**) represent the FCC phase and B2 phase, respectively. (**f**) The number density of microcracks in the Hf0 alloys. Error bars represent standard deviation. There were almost no grain boundary and phase boundary cracks in the Hf0.5 alloys. The purple and green arrows in (**a**) represent grain boundary cracks and phase boundary cracks, respectively.

The corresponding longitudinal cross-sections are shown in Figure 8. Figure 8a shows optical images of the cross-section of the fractured Hf0 alloys after tensile tests at 700 °C. Although there were hardly any large cracks observed on the lateral surface, numerous narrow and long microcracks located in the FCC phase can be found, and there are a few phase boundary microcracks (green arrow) when zooming in on the fracture end. For the lateral surface of the Hf0.5 alloys, almost no grain boundary or phase boundary cracks were observed on the surfaces. In contrast, only circle-like microvoids in the FCC phase with blunted edges were identified (Figure 8b), indicating that the addition of Hf can effectively prevent intergranular cracking of the alloy. In addition, due to the significant deformation of the Hf0.5 alloy, the FCC phase and B2 phase significantly elongated along the tensile direction.

The EBSD phase map and inverse pole figure shown in Figure 8c,d confirm that these microcracks (purple arrow) in the FCC phase were intergranular for Hf0 alloys. The appearance of intergranular cracks was consistent with its intergranular fracture surface (Figure 7a). Compared with the phase boundary microcracks, these microcracks at the grain boundary almost occupied the whole cross-section of the fractured specimen, as shown in Figure 8f. In the EBSD kernel average misorientation (KAM) map shown in Figure 8e, stress concentration near the grain boundary microcracks was observed, which was related to thermally activated grain boundary sliding and the accumulation of dislocations in their vicinity. It is worth mentioning that although there was a local stress concentration along the phase boundary, the number density of microcracks of the phase boundary was significantly lower than that of the grain boundary (Figure 8f). This indicates that the strength of the phase boundary was higher than that of the grain boundary and that the phase boundary can hinder the movement of dislocations at 700 °C.

Meanwhile, due to large residual stress in the fractured Hf0.5 alloy sample, the resolution rate of the EBSD of the lateral surface was extremely low. Therefore, the corresponding EBSD map of the Hf0.5 alloy is not provided in Figure 7. Based on its microstructure (Figure 2a), it can be inferred that the Hf-rich phases at grain boundaries and phase boundaries would pin grain and phase boundaries to delay the initiation and propagation of grain boundary and phase boundary cracks during high-temperature deformation, which was consistent with the phenomenon observed on the cross-section of the Hf0.5 alloy (Figure 8b), ensuring excellent tensile ductility.

## 4. Conclusions

In this work, six alloys with different minor Hf contents were designed from the initial composition of Al_17_Cr_10_Fe_36_Ni_36_Mo_1_ to study the effect of Hf on the microstructure and mechanical properties of the alloy. The main conclusions are summarized as follows:

1. With the increase in Hf content, the dual-phase structure with the FCC phase and B2 phase is almost unchanged while Hf is segregated near the grain boundary and phase boundary. In Al_17_Cr_10_Fe_35.5_Ni_36_Mo_1_Hf_0.5_ alloys, precipitation of the Hf-rich phase precipitated at grain boundaries and phase boundaries. In addition, the acicular Hf-rich phases with widths of 1~5 nm interlaced with each other and precipitated in the B2 phase were also observed.
2. At room temperature, with the increase in Hf content, the ultimate tensile strength and fracture strain of the alloys had little change. The Hf0.03 alloy with a moderate yield strength (867 MPa) exhibited the highest tensile strength (1533 MPa) and elongation to failure (18.6%). The Hf0.5 alloys with excellent tensile strength (1480 MPa) and elongation to failure (12.5%) exhibited the maximum yield strength (908 MPa).
3. At 700 °C, with the increase in Hf content, the alloy’s strength and plasticity increased first and then decreased. The Hf0.5 alloys demonstrated the best mechanical properties, with a yield strength of 816 MPa, tensile strength of 923 MPa, and elongation to failure of 42%. The strengthening effect of Hf on the alloy could be maintained at 600 °C, 800 °C, and 900 °C.
4. At different high temperatures, the Hf0.5 alloy exhibited a higher strength and ductility than the Hf0 alloys. The outstanding high-temperature strength of the Hf0.5 alloy was attributed to the precipitation strengthening of the Hf-rich phase precipitated in the inner of the B2 phase. The excellent high-temperature ductility of the Hf0.5 alloy was attributed to the pinning effect of the Hf-rich phase that existed at grain and phase boundaries to boundary sliding during the high-temperature deformation.

## Figures and Tables

**Table 1:** Mechanical test results of the five alloys with different Hf contents at 25 °C and 700 °C; σ_YS_, σ_UTS_, and ε_f_ represent the yield strength, tensile strength, and plastic strain, respectively.

Alloys	σ_YS_ (MPa)		σ_UTS_ (MPa)		ε_f_ (%)	
	25 °C	700 °C	25 °C	700 °C	25 °C	700 °C
Hf0	838	474	1404	535	10.8	8.7
Hf0.03	867	556	1533	620	18.6	9.0
Hf0.15	875	654	1475	704	11.9	13.0
Hf0.3	867	718	1496	787	13.7	27.0
Hf0.5	908	816	1480	923	12.5	42.0
Hf0.8	864	751	1443	849	9.5	20.0

**Table 2:** The exact values of yield strength (σ_YS_), ultimate tensile strength (σ_UTS_), and fracture elongation (ε_f_) tested at 700 °C for the Hf0.5 alloys.

Samples	σ_YS_ (MPa)	σ_UTS_ (MPa)	ε_f_ (%)
1#	793	908	45.3
2#	816	923	42.0
3#	778	904	44.6

**Table 3:** The exact values of yield strength (σ_YS_), ultimate tensile strength (σ_UTS_), and fracture elongation (ε_f_) tested from 25 °C to 900 °C for the Hf0.5 alloy and Hf0 alloy.

Temperature (°C)	σ_YS_ (MPa)		σ_UTS_ (MPa)		ε_f_ (%)	
	Hf0.5	Hf0	Hf0.5	Hf0	Hf0.5	Hf0
25	908	838	1480	1404	12.5	10.8
600	835	705	1145	921	31.7	11.3
700	816	474	923	535	42.0	8.7
800	426	221	465	256	80.7	39.7
900	205	111	223	130	77.6	100.7

## Data Availability

Data will be made available on request.
